# Association of the Heart Rate Variability Response to Active Standing with the Severity of Calcific Aortic Valve Disease: Novel Insights of a Neurocardiovascular Pathology

**DOI:** 10.3390/jcm11164771

**Published:** 2022-08-16

**Authors:** Jimena Rodríguez-Carbó, José M. Torres-Arellano, Nydia Ávila-Vanzzini, Rashidi Springall, Rafael Bojalil, Oscar Infante, Claudia Lerma, Juan Carlos Echeverría

**Affiliations:** 1 Posgrado en Ingeniería Biomédica, División de Ciencias Básicas e Ingeniería, Universidad Autónoma Metropolitana, Unidad Iztapalapa, Mexico City 09340, Mexico; 2 Departamento de Instrumentación Electromecánica, Instituto Nacional de Cardiología Ignacio Chávez, Mexico City 14080, Mexico; 3 Departamento de Consulta Externa, Instituto Nacional de Cardiología Ignacio Chávez, Mexico City 14080, Mexico; 4 Departamento de Inmunología, Instituto Nacional de Cardiología Ignacio Chávez, Mexico City 14080, Mexico; 5 Departamento de Atención a la Salud, División de Ciencias Biológicas y de la Salud, Universidad Autónoma Metropolitana, Unidad Xochimilco, Mexico City 04960, Mexico; 6 Departamento de Ingeniería Eléctrica, División de Ciencias Básicas e Ingeniería, Universidad Autónoma Metropolitana, Unidad Iztapalapa, Mexico City 09340, Mexico

**Keywords:** calcific aortic valve disease, heart rate variability, hierarchical partitioning, autonomic nervous system, active standing

## Abstract

The aim of this work was to obtain insights of the participation of the autonomic nervous system in different stages of calcific aortic valve disease (CAVD) by heart rate variability (HRV) analysis. Studying subjects with no valve impairments and CAVD patients, we also sought to quantify the independent contribution or explanatory capacity of the aortic valve echocardiographic parameters involved in the HRV changes caused by active standing using hierarchical partitioning models to consider other variables or potential confounders. We detected smaller adjustments of the cardiac autonomic response at active standing caused specifically by the aortic valve deterioration. The highest association (i.e., the highest percentage of independent exploratory capacity) was found between the aortic valve area and the active standing changes in the short-term HRV scaling exponent α_1_ (4.591%). The valve’s maximum pressure gradient echocardiographic parameter was present in most models assessed (in six out of eight models of HRV indices that included a valve parameter as an independent variable). Overall, our study provides insights with a wider perspective to explore and consider CAVD as a neurocardiovascular pathology. This pathology involves autonomic-driven compensatory mechanisms that seem generated by the aortic valve deterioration.

## 1. Introduction

Calcific aortic valve disease (CAVD) is manifested as aortic valve sclerosis (AVSc) at an early stage, or as aortic valve stenosis (AVSt) that may require surgical treatment [1]. CAVD prevalence increases with age: AVSt goes from 0.2% in 50–59-year-old patients to 9.8% in 80–89-year-old patients [2], while AVSc increases from 25% in 65-year-old patients up to 40% in 75-year-old patients [3].

AVSt occurs at the end of an inflammatory process caused by endothelial damage owing to mechanical stress and lipid penetration of leaflets that lead to fibrosis, thickening, and finally calcification [4,5,6]. Calcific AVSt causes increased leaflet stiffness and a narrowed aortic valve orifice that increases the pressure gradient across the valve. CAVD has the prolonged subclinical period of AVSc during which, despite some calcification occurring within the valve, the aortic valve function parameters are still measured within a normal range [2].

Whereas AVSc is not always diagnosed, most patients with AVSt are usually identified when a cardiac auscultation reveals a systolic murmur or owing to the presence of indicative symptoms [1]. Symptomatic patients in the AVSt stage have very poor prognosis [2]. Therefore, exploring and developing different tools and frameworks that help to improve our understanding of CAVD, as well as allowing an early diagnosis, are still important [7]. Echocardiography has become the standard for evaluation of CAVD. The primary hemodynamic parameters recommended in different guidelines for a clinical evaluation of CAVD are maximum aortic velocity (Vmax), aortic valve area (AVA), aortic valve area indexed to the body surface area (AVAi), maximum pressure gradient (PGmax), and mean pressure gradient (PGmean) [8,9,10].

The analysis and interpretation of heart rate variability (HRV), the beat-to-beat variation in either the instantaneous heart rate or the R-R interval, has become a recurrent clinical and investigational tool, in particular for cardiovascular diseases. These temporal fluctuations are widely believed to reflect changes in the cardiac autonomic regulation [11]. Even though the definition of CAVD and its clinical assessment is focused on the deterioration of the aortic valve, there are several other mechanisms that are presumed to be involved in this disease, such as changes in the cardiac autonomic function. Using HRV analysis and muscle sympathetic nerve activity measurements, Arslan et al. [12], Jung et al. [13], Dumontier et al. [14], Echeverria et al. [15], and Torres-Arellano et al. [16] have reported differences in cardiac autonomic function at different stages of CAVD. These studies have documented evidence of an elevated sympathetic activity in CAVD patients. Echeverria et al. [15] and Torres-Arellano et al. [16] specifically reported a difference in the cardiac autonomic response to the active standing in both patients with AVSc and AVSt; the difference in the HRV parameters and, therefore, the particular response to this orthostatic stimulus were found to be diminished according to the severity of CAVD.

Only a few studies have searched for an association between the echocardiographic valve function parameters and the HRV indices in CAVD. Arslan et al. [12] calculated the bivariate correlations between PGmean, PGmax and AVA and the HRV indices in supine position, but none were significant. This absence of correlations might be explained by the inclusion of only AVSt patients in a mild and moderate but not severe stage and because they did not include patients with AVSc. A similar result was found in [13] between PGmax and HRV indices, where the absence of correlations could be attributed to the inclusion of only severe AVSt patients and whose HRV indices were only calculated during supine position.

Given the above reported differences in the autonomic response to active standing in patients with different stages of CAVD, we hypothesized that the echocardiographic parameters of the aortic valve function are independently associated with changes provoked by active standing in the HRV indices once several other variables that can also modify such indices are considered. The aim of this work was then to quantify among subjects not having impairments in the aortic valve and CAVD patients the independent contribution or explanatory capacity of such echocardiographic parameters in the HRV changes provoked by active standing.

## 2. Materials and Methods

### 2.1. Subjects and Study Protocol

A cross-sectional study was carried out at the National Institute of Cardiology “Ignacio Chávez” at Mexico City including participants having normal aortic valve (NAV), AVSc and AVSt. These 3 groups were chosen to include different stages of CAVD.

Exclusion criteria were: coronary ischemic disease, renal function alterations, liver disease, recent infections (last month), influenza vaccination within the last six months, autoimmune diseases (e.g., lupus, arthritis, or others), or moderate or significant injury in the mitral or tricuspid valves. For the NAV and AVSc groups, volunteers who were considered healthy were recruited after an invitation alongside staff and patient’s relatives of the Institute. They had no known comorbidities and were not taking any medication. Afterwards, an echocardiogram was performed on each volunteer, and they were classified within the NAV or AVSc group. The AVSt group comprised patients previously diagnosed with calcific aortic valve stenosis, being candidates for the elective valve replacement program, who were recruited during a follow-up visit to the outpatient’s clinic of the Institute. Patients with bicuspid aortic valve or AVSt rheumatic etiology were excluded.

We obtained a group of 22 NAV subjects, 73 AVSc and 32 AVSt patients. Any previous history of hypertension, diabetes, dyslipidemia, alcoholism, and smoking was obtained from a questionnaire of each participant and corroborated by the clinical records in AVSt patients.

Anthropometric measures, oscillometric blood pressure, and resting 12-lead standard electrocardiogram (ECG) were obtained. A second continuous ECG recording (using a sample rate of 250 Hz) was performed with a chest band (BioHarness 3.0, Zephyr Technology, Annapolis, MD, USA) [17] while participants remained in a supine position for 10 min, followed by active standing for another 10 min. At supine position, participants were also asked to lie with legs uncrossed and hands by their sides. Then, a blood sample was taken, and finally, a 2D transthoracic echocardiogram was performed.

### 2.2. Echocardiographic Assessment

One specialist measured the echocardiographic parameters by two-dimensional Doppler, employing a commercial machine (iE33, Philips Healthcare, Bothell, WA, USA). Using a pulsed-wave Doppler recording, the following echocardiographic parameters were obtained: maximum aortic valve transvalvular velocity (m/s, Vmax) and mean and maximum mean pressure gradient (mmHg, PGmean and PGmax), aortic valve area (cm^2^, AVA), aortic valve area indexed to body surface area (cm^2^/m^2^, AVAi), left ventricular ejection fraction (%, LVEF), left ventricular mass (g, LVM), left ventricular mass indexed to body surface area (g/m^2^, LVMi), and relative wall thickness (RWT). The patients were classified within a study group (NAV, AVSc, and AVSt) according to current guidelines [8].

### 2.3. Electrocardiogram Recording and HRV Indices

**Figure 1:**
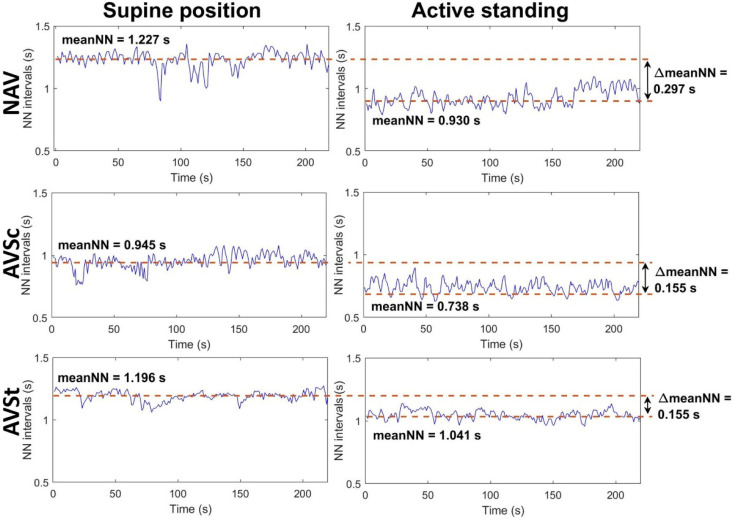
Example of HRV time series from one NAV subject (upper panels), one AVSc patient (middle panels) and one AVSt patient (lower panels) while they were in supine position (left panels) and active standing (right panel). The NAV subject is a 46-year-old man with a BMI of 25.565 kg/m^2^, a Vmax of 1.2 m/s, an AVA of 3.9 cm^2^, an AVAi of 2.02 cm^2^/m^2^, a PGmean of 2 mmHg, a PGmax of 5 mmHg and an LVEF of 60%. The AVSc patient corresponds to a 30-year-old-man with a BMI of 26.3 kg/m^2^, a Vmax of 1.05 m/s, an AVA of 4.2 cm^2^, an AVAi of 2.2 cm^2^/m^2^, a PGmean of 2 mmHg, a PGmax of 4 mmHg and an LVEF of 68%. The AVSt patient is a 53-year-old man with a BMI of 30.191 kg/m^2^, a Vmax of 5.6 m/s, an AVA of 0.5 cm^2^, an AVAi of 0.25 cm^2^/m^2^, a PGmean of 57 mmHg, a PGmax of 97 mmHg and an LVEF of 55%. MeanNN: mean value of all NN intervals in the time series, NAV: normal aortic valve, AVSc: aortic valve sclerosis, AVSt: aortic valve stenosis. ΔmeanNN: the difference between meanNN values at the supine position minus values at active standing.

About 5 min of the chest-band ECG recordings in each position (supine position and active standing) were selected to obtain 300 heart-period intervals or HRV time series. The ECG QRS complexes were identified by a second derivative algorithm [18], followed by manual inspections to remove artifacts and ectopic beats obtaining only RR intervals from sinus rhythm origin (NN intervals). HRV indices were assessed from such 300 consecutive RR intervals obtained at the supine position and active standing. Figure 1 shows an example of HRV time series from one NAV subject (upper panels), one AVSc subject (middle panels) and one AVSt patient (lower panels). The left panels depict the time series of each subject collected in supine position, while the right ones show the times series in active standing.

The following HRV time-domain indices were calculated for each time series: mean NN (average value of all RR intervals), SDNN (standard deviation of all NN or RR intervals), pNN20 (percentage of successive RR intervals with differences greater than 20 ms), and RMSSD (the square root of the mean squared differences in successive NN intervals) [19]. For estimating frequency domain HRV indices, each time series was resampled using a linear interpolation method at three samples per second, and then the power spectrum density was estimated by Welch’s periodogram. The mean spectral power was obtained for the low frequency (LF) band (0.04 to 0.15 Hz) and the high frequency (HF) band (0.15 to 0.4 Hz) and the ratio between them (LF/HF). The LF and HF indices were transformed to normalized units (LFn and HFn) [20]. Whereas LF is considered to reflect the cardiac response to both sympathetic and parasympathetic activities, HF is regarded as a reliable parameter of the vagal cardiac influence. The fractal scaling exponent α_1_ was calculated for each original HRV time series by applying detrended fluctuation analysis within the short-range of scales (4 to 11 RR intervals) [21]. This index indicates the irregularity and directionality of time series, which are thought to be influenced by the cardiac-autonomic interplay. Sample entropy (SampEn) [22] was also obtained, which indicates the regularity of the times series. For all these indices, the difference (Δ) between values obtained at the supine position minus values at active standing was estimated to assess the magnitude of change. The HRV indices estimation was performed with ad hoc validated computer programs developed in MATLAB (MathWorks, Inc., Natick, MA, USA) [23]. For SampEn and α_1_, algorithms obtained from Physionet [24] were used.

### 2.4. Breathing Frequency

Mean breathing frequency (MBF) was obtained using the respiratory movements data (sample rate of 25 Hz) from the same chest band from which the ECG was registered. From these time series, the frequency spectrum was calculated using the Fourier Transform, and the frequency that had the maximum power value was chosen as the MBF.

### 2.5. Blood Samples Collection and Analysis

We collected 10 mL blood samples from each participant. The samples were centrifuged at 3000 rpm for 15 min, at 4 °C, and stored in aliquots, at −76 °C. Serum concentrations of matrix metalloproteinase-1 (MMP1), matrix metalloproteinase-2 (MMP2), matrix metalloproteinase-3 (MMP3), matrix metallopeptidase-9 (MMP9), tissue inhibitor of metalloproteinase-1 (TIMP1), interleukin 4 (IL-4), interleukin 6 (IL-6), interleukin 10 (IL-10), interferon gamma (IFN-γ), tumor necrosis factor alpha (TNF-α), tumor growth factor beta (TGF-β), leukotriene B4 (LTB4), lipoxin A4 (LXA4), endothelin 1 (ET-1), prostaglandin E2 (PGE-2), and resolving D1 (RvD1) were measured by enzyme-linked immunosorbent assays with commercial kits (R&D Systems) following the manufacturer’s instructions. The serum levels of C-reactive protein (CRP) were determined by nephelometry (Beckman Coulter).

Markers of the inflammatory process were divided into groups depending on their function: anti-inflammatory mediators (IL-4, IL-10, and TGF-β), lipidic resolving mediators (LXA4 and RvD1), lipidic inflammatory mediators (LTB4 and PGE-2) and inflammatory mediators (CRP, IFN-γ, IL-6, TNF-a, ET-1, and IL-12). Two other groups of variables were created, the matrix metalloproteinases (MMPs) and TIMP1, and the ratio between the MMPs and TIMP1 (MMP1/TIMP1, MMP2/TIMP1, MMP3/TIMP1, and MMP9/TIMP1).

The biochemical parameters concentration of serum glucose, hemoglobin, cholesterol, and triglycerides were also measured.

### 2.6. Study Variables

**Figure 2:**
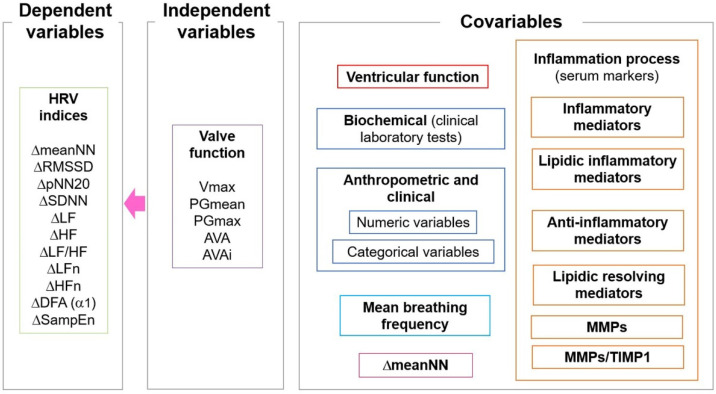
Conceptual framework of the studied variables. Vmax: maximum aortic valve transvalvular velocity; PGmean: mean pressure gradient, PGmax: maximum pressure gradient; AVA: aortic valve area, AVAi: aortic valve area indexed to body surface area; Δ: the difference between values of heart rate variability (HRV) indices at the supine position minus values at active standing; meanNN: mean value of all NN intervals (RR intervals from sinus rhythm); SDNN: standard deviation of all NN intervals; RMSSD: root mean squared of the successive differences; pNN20: percentage of successive NN intervals with differences greater than 20 ms; LF: low-frequency band spectral power; HF: high-frequency band spectral power, n.u.: normalized units; LF/HF: ratio between low-frequency and high frequency spectral powers; α_1_: short-term scaling exponent, SampEn: sample entropy; MMPs: matrix metalloproteinases; TIMP1: tissue inhibitor of metalloproteinase-1.

Figure 2 shows a diagram representing the conceptual framework of the studied variables. The HRV indices were here considered as the dependent variables, assessed as the magnitude of change (Δ) measured by the arithmetic difference in indices between supine position—active standing. The main independent variables were the valve function parameters evaluated by echocardiography. All other independent variables considered covariables in our analysis include the echocardiographic parameters of the ventricular function (FEVI, LVM, LVMi, and RWT), biochemical concentration parameters, the mediators of the inflammatory process (as described in the previous section), and anthropometric and clinical variables: age, body mass index (BMI), MBF, systolic blood (SBP), diastolic blood pressure (DBP), sex, medication intake (i.e., being under any pharmacological treatment), smoking, hypertension, and diabetes. The magnitude of change in the mean cardiac period (ΔmeanNN) between supine position and active standing, as illustrated in Figure 1, was also considered as a covariable [25].

### 2.7. Statistical Analysis

For continuous numeric variables, an Anderson–Darling test [26,27] was applied to determine normal distributions. Variables of the three groups with normal distribution are reported as mean ± standard deviation and were compared among groups using ANOVA. For variables not showing a normal distribution, the results are reported as median (percentile 25, percentile 75), and were compared among groups by a Kruskal–Wallis test. For categorical variables, the results are reported as absolute values (percentage) and were compared using a chi-square test.

A post hoc bivariate analysis was considered (NAV vs. AVSc, NAV vs. AVSt and AVSc vs. AVSt) by using a Mann–Whitney test, Student’s t test or a chi-square test, depending on whether or not the variables showed a normal distribution or were categorical. The significance level was adjusted using a Bonferroni correction for multiple comparisons [28].

### 2.8. Hierarchical Partitioning

**Figure 3:**
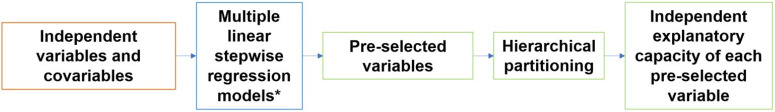
Schematic representation of hierarchical partitioning analysis applied to each of the 11 dependent variables (HRV indices). The pre-selected variables were distinctive for each of the 11 HRV indices. * A multiple linear stepwise regression model was applied for each category of independent variables and covariables as described in Figure 2.

Figure 3 shows a schematic representation of the hierarchical partitioning analysis. Given the combined large number of independent variables and covariables (or confounding measurements), multiple linear stepwise regression models without interactions were initially performed to pre-select variables that were then considered for the hierarchical partitioning within each category in Figure 2 (i.e., valve function, ventricular function, biochemical, anthropometric/clinical, MBF, markers of the inflammatory process and ΔmeanNN). By considering that this process was applied to each of the 11 dependent variables (HRV indices), the pre-selected variables were then distinctive for each dependent variable.

Hierarchical partitioning analysis was used to estimate the independent explanatory capacity of each independent variable (valve function) on the dependent variables (the Δ, i.e., the difference between values of HRV indices at the supine position minus values at active standing) while also taking into consideration all the other covariables or confounding measurements shown in the diagram of Figure 2 that could also elicit collinear effects on the dependent variables. The actual process of hierarchical partitioning involves computing, by averaging, the improvement in the goodness of fit (here, the R² value) of all models that include a particular independent variable or covariable as compared with the corresponding equivalent model that does not consider such independent variable or covariable [29]. For hierarchical partitioning, the “*hier.part*” function in the “*hier.part*” package was used (R software, version 3.6.0) [30].

The “*hier.part*” program was run 10 times by changing the order in which the predictor variables were entered for the case of the dependent variable Δα_1_, which involved more than 9 predicting or independent variables. The independent contributions were then estimated by averaging the results of all those repetitions.

## 3. Results

Table 1 shows the anthropometric and clinical characteristics of the study groups. Compared with the NAV subjects, patients in the groups AVSc or AVSt were older, had higher systolic blood pressure as well as MBF, and such groups included more cases with hypertension, diabetes, smoking, and prescribed medications. There were no significant differences in other variables.

For the echocardiographic parameters regarding the valve function (Table 2), the results were consistent with the criteria used for diagnosis. The principal differences in these parameters were identified in the AVSt group: larger Vmax, PGmean, and PGmax; smaller AVA and AVAi.

The echocardiographic parameters regarding the ventricular function of the AVSt group were also different from the other two groups (Table 3). LVMi in the AVSc groups also differs from the NAV group.

Table 4 shows the descriptive values of the HRV indices in each group of subjects. For ΔmeanNN, ΔHF, ΔLF/HF, ΔHFn, ΔLFn, and Δα_1_, the values from the AVSt group were smaller and different from the other two groups. Additionally, for ΔLF/HF and ΔLFn, the AVSc groups show differences with the NAV group. For ΔLF, the groups AVSt and AVSc were different from the NAV group. In general, for the HRV indices that show differences between the groups, their magnitude values changes (Δ) diminish with the disease progress, from NAV to AVSt.

Before applying the hierarchical partitioning, multiple linear stepwise regression models were considered to select the variables in each category of independent variables and covariables. Table S1 in the Supplementary Materials shows the full description of selected variables for the time domain HRV indices.

In summary, for ΔpNN20, the independent variable selected was PGmean, and the covariables were age, albumin, CRP, medication intake and ΔmeanNN. For ΔmeanNN, considered as a dependent variable, the independent variable selected was PGmean, and LVMi, glucose, triglycerides, ET1, age, medication intake and MBF were the covariables. Although ΔRMSSD did not have a valve function parameter selected as independent variable, age, medication intake and ΔmeanNN were the covariables. ΔSDNN also did not have an independent valve function parameter variable, but it had CRP, IFN-γ, BMI, and medication intake as the covariables selected in the multiple linear stepwise regression model.

Table S2 in the Supplementary Materials shows the full description of the selected variables for the frequency domain HRV indices according to the multiple linear stepwise regression models. In summary, ΔLF did not have a valve function parameter selected as independent variable, but it had RWT, CRP, TIMP1 and BMI as the covariables. For ΔHF, the independent variable selected was PGmean, and the covariables were SBP and ΔmeanNN. For ΔLF/HF, the independent valve function parameter variable was PGmax, and the covariables were RWT, SBP, medication intake, MBF and ΔmeanNN. For ΔLFn, the independent valve function parameter variable was PGmean, and the covariables were LVM, glucose, triglycerides, ET1, SBP, DBP, MBF, and ΔmeanNN. For ΔHFn, the independent valve function parameter variable was PGmean, and the covariables were LVM, glucose, triglycerides, ET1, SBP, DBP, MBF and ΔmeanNN.

Table S3 in the Supplementary Materials shows the full description of selected variables for the nonlinear HRV indices according to the multiple linear stepwise regression models. In summary, for Δα_1_, the independent valve function parameter variable was AVA, and the covariables were RWT, LVEF, triglycerides, ET1, IL-4, SBP, age, medication intake MBF and meanNN.

For ΔSampEn, the independent variable was PGmean, and the covariables were MMP2/TIMP1, SBP and ΔmeanNN.

The most present covariables that were selected in the multiple linear stepwise regression models were ΔmeanNN (in 8 out of 10 models), medication intake (in 6 out of 11 models), SBP (in 6 out of 11 models), MBF (in 5 out of 11 models), and age (in 4 out of 11 models).

Table 5 shows the percentage of independent exploratory capacity, as provided by the hierarchical partitioning analysis for the independent valve function parameter variables. The table also shows the specific pre-selected covariables in the models for each dependent HRV variable and the overall R² of such analysis. The independent variable (valve function echocardiographic parameter) most selected was PGmean (in 6 out of 8 dependent HRV variables), while the independent variable with the highest percentage of independent contribution was AVA with Δα_1_ (4.591%); Δα_1_ was also the dependent variable with the highest number of covariables included in its stepwise regression model.

Figures S1–S11 in the Supplementary Materials show the percentage of independent explanatory capacity for all the independent variables and covariables in descending order in relation to all dependent variables. Such figures also show the R² of the final combined model assessed by the hierarchical partitioning analysis.

## 4. Discussion

The main findings of this work are the associations between the echocardiographic parameters of the aortic valve and the differences in the cardiac autonomic response to active standing as suggested by most of the changes (Δ) of the HRV indices studied. These associations remained significant even when considering other covariables and their differences among studied groups such as age, the changes in meanNN, medication intake, and several others.

We also observed a smaller autonomic response or adjustment with the severity of the valve deterioration because the changes (Δ) to active standing in the HRV indices were significantly smaller in the case of either smaller AVA as well as AVAi or higher Vmax, PGmean and PGmax.

Using the hierarchical partitioning analysis, we were also able to estimate the percentage of independent exploratory capacity for the echocardiographic parameters of the aortic valve. This percentage indicates the changes in the autonomic-related dependent variables to active standing that can be specifically attributed to the valve deterioration.

The highest association (i.e., the highest percentage of independent exploratory capacity) was found between AVA and Δα_1_ with a 4.591%, but the echocardiographic valve parameter that was present in more models assessed by hierarchical partitioning was the PGmean (in six out of eight HRV indices models that included a valve function parameter).

In addition to the echocardiographic valve parameter AVA, Δα_1_ was in fact the HRV index with more covariables chosen other than ΔmeanNN (i.e., nine covariables) that exert influence on its changes to active standing. This could explain why it is useful and extensively used to differentiate and explore different pathologies [31].

The hierarchical partitioning analysis also allowed us to identify other relevant covariables to explain the changes in the HRV indices to active standing. The covariable that showed the highest percentage of independent explanatory capacity was in fact ΔmeanNN (25.174% in the hierarchical partitioning analysis of ΔRMSSD). As expected by previous publications [32,33], it was also selected in most models (for 8 out of 10 HRV dependent variables). The other most chosen covariables were medication intake, SBP, MBF and age.

In the CAVD patients, the smaller changes in HRV found (smaller adjustments) can be attributed to a modified response to the ANS modulation because we used a controlled stimulus (active standing) for which the expected response has been well studied. Active standing usually causes between 500 and 800 milliliters of blood volume to shift from the upper to the lower parts of the body, and this redistribution causes a drop in both venous return and right atrium pressure, reducing the stroke volume and eventually the blood pressure [34]. It is generally assumed that the physiological detection of this redistribution occurs via the baroreceptors that respond to the tension drop in the blood vessel walls (owing to the decreased blood pressure), which causes a decreased baroreceptors firing rate to the solitary nucleus in the medulla [34]. This reduced firing rate from the baroreceptor nerves inhibits the efferent parasympathetic activity and stimulates the sympathetic one. The cardiovascular responses to these stimuli are increments in heart rate, SBP, vasoconstriction and cardiac output [35]. It is then considered that these responses and the changes seen in the HRV indices owing to such change in position show a vagal withdrawal and a greater sympathetic cardiac response [36,37,38]. Taking into consideration this autonomic response, the smaller changes or limited adjustments in the HRV indices of our CAVD patients indicate a smaller vagal withdrawal and a reduced response to the sympathetic activation. This could then reflect an autonomic-driven compensatory mechanism.

As just described, the active standing test provokes a vagal withdrawal and an increase in the sympathetic cardiac response that leads to a higher heart rate and smaller meanNN. This decrease in the meanNN was limited in our patients with CAVD because we found smaller changes or Δ for cases with a higher valve deterioration. As indicated before, the valve function parameter (i.e., the independent variable) associated with the ΔmeanNN was PGmean (the higher the mean valve pressure gradient, the smaller the change in meanNN during active standing). This association could therefore suggest that the baroreceptors of the aortic arch (next to the aortic valve) detect these changes in the pressure gradient and, as a result, respond to them by limiting the cardiac sympathetic response when there is a change in position, which would limit the increase in heart rate and, therefore, the increase in blood pressure.

These smaller adjustments to active standing have also been observed in patients with hypertension [39,40], which occurs as a common comorbidity in patients with CAVD (specially AVSt), and it is associated with a worse outcome [41,42,43,44]. In our patients, those with AVSt had a higher incidence (50%) of hypertension than the NAV group (9%). Patients with hypertension have as well a higher basal cardiac sympathetic activity compared to subjects without hypertension [45,46,47]. Although the presence of hypertension (dichotomous variable) was not selected as a covariable in our stepwise models and therefore not considered in the hierarchical partitioning analysis, the SBP was included in 6 out of 11 HRV indices models. The smaller change in ΔmeanNN was not associated with the SBP but it was related to PGmean, which could again reinforce the idea that the baroreceptors located in the aortic arch respond differently owing to the changes in the pressure gradient of the aortic valve.

Another compensatory mechanism that could be involved is the one in which the patients with CAVD have a sympathetic hyperactivity that has been identified through muscle sympathetic nerve activity (MSNA) [14] and the frequency domain HRV indices (higher LFn and lower HFn) [15,16] when compared to subjects without CAVD. This sympathetic overactivity could also limit the response to active standing. In a previous study [48], authors found an indirect relationship between the sympathetic activity during rest and responses that are mediated by the baroreflex and those that are not. They identified that the sympathetic response diminished when the subjects showed higher levels of sympathetic activity during rest measured with MNSA. In our subjects, this restricted adjustment range could also be responsible for limiting the increase in heart rate when changing position and, correspondingly, for limiting the reduction in meanNN.

The two mechanisms considered (i.e., a reduced autonomic adjustment to meet the hemodynamic demands without an overshoot, or a restricted autonomic response owing to the baseline saturated control of the chronic sympathetic overactivity) could be the cause of the smaller changes in meanNN observed here. Given this, the relevant participation that the ΔmeanNN had as a covariable for the other HRV dependent indices (it was included in almost all the other indices except for ΔSDNN and ΔLF) must be mentioned and considered. Previous studies have disclosed that there exists an unequivocal relationship between the meanNN and other HRV indices [33,49], which was also seen here with the multiple linear stepwise regression models where ΔmeanNN was selected in 8 out of 10 models. Meanwhile, it has been reported as well that in CAVD subjects, there seem to be no differences in the meanNN during supine position [12,14,15,16]. Therefore, it can be considered that the difference in the ΔmeanNN seen here in the CAVD patients is not caused by having a different original operating point in relation to meanNN, but rather due to a different cardiac autonomic response. Accordingly, such limitation in the change in meanNN should be involved in the lower response to active standing manifested by most of the HRV indices.

The association between autonomic cardiac modulation and CAVD at different stages suggests potential clinical implications to be explored in future investigations. Sympathetic predominance could have a role in the early stages of CAVD (AVSc and mild AVSt) due to a less efficient vagal modulation of a low-grade inflammation [15,50]. Early detection and preventive measures for CAVD may be benefited by a more comprehensive study of patients at risk of CAVD including assessment of cardiac autonomic modulation. Similarly, the autonomic cardiac modulation may be different between patients with rapid or rather slow progression of AVSt, or it may also be linked to higher risk of poor clinical outcomes after aortic valve replacement. Therefore, searching for better predictive markers of progression or negative post-surgery outcomes through non-invasive HRV analysis is worth being considered.

## 5. Study Limitations and Further Work

Given our study design, we are not able to infer the causality between the independent variables and covariables and the HRV indices. Our study subjects had comorbidities (i.e., hypertension and diabetes), different ages (CAVD is considered an age dependent disease) and medication intake (due to the comorbidities), which can clearly influence the HRV indices. However, the approach followed here, based on hierarchical partitioning, allowed us to take into consideration and quantify the particular effect of those different variables among groups while still identifying the independent explanatory capacity of the echocardiographic parameters of the aortic valve on the HRV indices. This is the reason why such variables were included in several of our final models (as indicated in Table 5) with the particular effects reported in the Supplementary Materials.

Other strategies such as larger heterogeneous samples with stratified analysis may be useful in further studies to increase the generalization of the present findings. Our study sample had missing data in certain covariables. The sample size was thus explicitly described.

Although hierarchical partitioning models are very useful in finding an exact value of association between variables, the way that association is calculated depends on the R² obtained from those models, so changing the number of variables added into the models may change the percentage assessed for the independent explanatory capacity. Additionally, the models used here were based on a linear regression approach, so another type of model could be used as well to consider the potential non-linear relationship between variables.

We did not explore here the possibility of transforming variables to warrant homoscedasticity or normal distribution, which could favor linear correlations. It is then also required to study the effect that heteroscedasticity could have in the hierarchical partitioning analysis and specifically to the analysis involving HRV indices.

## 6. Conclusions

The hierarchical partitioning analysis allowed us to find the independent explanatory capacity of the valve parameters in relation to the adjustments of the HRV indices during active standing. This framework led us to detect a smaller adjustment of the cardiac autonomic response during active standing, caused specifically by the valve deterioration present in CAVD, mainly in AVSt.

The highest percentage of independent explanatory capacity for the echocardiographic parameters was found between AVA and Δα_1_; nonetheless, PGmean was the valve echocardiographic parameter most recurrent in the hierarchical partitioning models. The hierarchical partitioning analysis also enabled us to identify and quantify the effect, and their differences among groups, of other important covariables such as the ΔmeanNN, SBP and MBF. With these results, we were able to suggest additional pathophysiological mechanisms potentially involved in the CAVD progress. These mechanisms were the existence of a higher basal sympathetic activity with limited adjustment range, and the restriction of the cardiac response to the sympathetic activity owing to a higher-pressure gradient in the aortic valve by means of the baroreceptors.

Overall, our study distinctly revealed insights with a wider perspective to explore and consider calcific aortic valve disease as a neurocardiovascular pathology. This pathology involves autonomic-driven compensatory mechanisms that seem generated by the aortic valve deterioration in itself.

## Figures and Tables

**Table 1:** Anthropometric and clinical values of participants. Data are shown as absolute value (percentage), mean ± standard deviation, or median (percentile 25, percentile 75).

Variable	NAV (*n* = 22)	AVSc (*n* = 73)	AVSt (*n* = 32)	*p* Value
Age (years)	41.3 ± 7.9	45.3 ± 9.3	63.3 ± 6.6 ^°	<0.001
Female	10 (45%)	40 (55%)	11 (34%)	0.150
BMI (kg/m^2^)	25.9 (24.9, 29.4)	27.0 (24.9, 30.3)	28.2 (26.7, 32.2)	0.141
DBP (mmHg) ^&^	78 (70, 80)	78 (70, 81)	80 (70, 83)	0.478
SBP (mmHg) ^&^	110 (108, 118)	116 (110, 123)	136 ± 21.4 ^°	<0.001
MBF (Hz)	0.27 ± 0.05	0.27 (0.22, 0.31)	0.33 ± 0.08 ^°	0.002
Medication intake ^&^	6 (27%)	18 (25%)	24 (75%) ^°	<0.001
Hypertension	2 (9%)	4 (5%)	16 (50%) ^°	<0.001
Smoking	6 (27%)	26 (36%)	12 (38%)	0.714
Diabetes	0 (0 %)	2 (3%)	7 (22%) °	<0.001

^ Comparing with NAV (*p_a_* < 0.017); ° Comparing with AVSc (*p_a_* < 0.017). *p_a_*: adjusted value of *p* according to the Bonferroni correction for multiple comparisons. NAV: normal aortic valve; AVSc: aortic valve sclerosis; AVSt: aortic valve stenosis; BMI: body mass index; SBP: systolic blood pressure; DBP: diastolic blood pressure; MBF: mean breathing frequency. Medication intake: being under any pharmacological treatment. ^&^ In the variables DBP and SBP, the total number of subjects was 107 (NAV = 21, AVSc = 57 and AVSt = 29); in Medication intake the total number of subjects was 125 (NAV = 22, AVSc = 71 and AVSt = 32).

**Table 2:** Parameters evaluated from the echocardiogram that depict the valve function. Data are shown as mean ± standard deviation, or median (percentile 25, percentile 75).

Variable	NAV	AVSc	AVSt	*p* Value
Vmax (m/s) *n* = 126	1.2 ± 0.3 *n* = 22	1.3 ± 0.2 *n* = 72	4.4 ± 1.2 ^° *n* = 32	<0.001
PGmean (mmHg) *n* = 126	3 (2, 3) *n* = 22	3 (2, 4) *n* = 72	41 (23, 71) ^° *n* = 32	<0.001
PGmax (mmHg) *n* = 126	5.3 ± 2.2 *n* = 22	6 (4, 7) *n* = 73	69 (37.2, 114.7) ^° *n* = 31	<0.001
AVA (cm^2^) *n* = 125	4.1 ± 0.2 *n* = 21	4.1 (4, 4.3) *n* = 72	0.6 (0.4, 1.3) ^° *n* = 32	<0.001
AVAi (cm^2^/m^2^) *n* = 122	2.2 ± 0.2 *n* = 21	2.3 ± 0.3 *n* = 72	0.4 (0.3, 0.7) ^° *n* = 29	<0.001

^ Comparing with NAV (*p_a_* < 0.017); ° Comparing with AVSc (*p_a_* < 0.017). *p_a_*: adjusted value of *p* according to the Bonferroni correction for multiple comparisons. NAV: normal aortic valve; AVSc: aortic valve sclerosis; AVSt: aortic valve stenosis; Vmax: maximum aortic valve transvalvular velocity; PGmean: mean pressure gradient; PGmax: maximum pressure gradient; AVA: aortic valve area; AVAi: aortic valve area indexed to body surface area.

**Table 3:** Parameters evaluated from the echocardiogram that depict the ventricular function. Data are shown as mean ± standard deviation, or median (percentile 25, percentile 75).

Variable	NAV	AVSc	AVSt	*p* Value
LVEF (%) *n* = 127	61.9 ± 6.4 *n* = 22	62.3 ± 6.6 *n* = 73	55 (51, 60) ^° *n* = 32	<0.001
LVM (g) *n* = 76	98 (86, 105) *n* = 16	117 (96, 155.7) *n* = 45	216.9 ± 67.1 ^° *n* = 15	<0.001
LVMi (g/m^2^) *n* = 76	54.7 ± 12.1 *n* = 16	65 (56.7, 77) ^ *n* = 45	119.8 ± 34.7 ^° *n* = 15	<0.001
RWT *n* = 76	0.4 ± 0.1 *n* = 16	0.4 ± 0.1 *n* = 45	0.5 ± 0.2 ^° *n* = 15	<0.001

^ Comparing with NAV (*p_a_* < 0.017); ° Comparing with AVSc (*p_a_* < 0.017). *p_a_*: adjusted value of *p* according to the Bonferroni correction for multiple comparisons. NAV: normal aortic valve; AVSc: aortic valve sclerosis; AVSt: aortic valve stenosis; LVEF: left ventricular ejection fraction; LVM: left ventricular mass; LVMi: left ventricular mass indexed to body surface area; RWT: relative wall thickness.

**Table 4:** Magnitude (Δ) of change in heart rate variability indices in response to active standing. Data are shown as mean ± standard deviation, or median (percentile 25, percentile 75). The *n* specifies the number of subjects in each group.

Variable	NAV (*n* = 22)	AVSc (*n* = 73)	AVSt (*n* = 32)	*p* Value
ΔpNN20 (%)	8.9 ± 7.3	9.9 ± 9.0	5.4 ± 10.2	0.060
ΔmeanNN (s)	0.2 ± 0.1	0.2 ± 0.1	0.1 (0.0, 0.1) ^°	0.001
ΔRMSSD (ms)	10.9 (7.0, 16.8)	14.4 (4.2, 27.5)	6.6 (−2.7, 18.6)	0.049
ΔSDNN (ms)	1.7 (−5.9, 13.9)	6.7 (−6.9, 23.7)	4.7 (−7.1, 18.6)	0.499
ΔLF (ms^2^)	−612.5 (−827.2, −93.3)	−26.1 (−433.4, 249.3) ^	22.2 (−118.2, 181.9) ^	0.001
ΔHF (ms^2^)	130.5 (36.7, 311.5)	152 (58.6, 406.1)	48.0 (−14.0, 90.0) ^°	0.003
ΔLF/HF	−5.8 ± 4.9	−3.0 (−6.6, −1) ^	−0.7 (−3.7, 0.5) ^°	0.001
ΔHFn (n.u.)	30 ± 19.3	19.6 ± 17.1	5.3 (−2.3, 14) ^°	<0.001
ΔLFn (n.u.)	−30.2 ± 19.3	−19.5 ± 17.1^	−5.1 (−14, 2.3) ^°	<0.001
Δα_1_	−0.4 ± 0.2	−0.3 ± 0.3	−0.1 ± 0.3 ^°	<0.001
ΔSampEn	0.3 ± 0.5	0.3 ± 0.4	0.1 ± 0.4	0.139

^ Comparing with NAV (*p_a_* < 0.017); ° Comparing with AVSc (*p_a_* < 0.017). *p_a_*: adjusted value of *p* according to the Bonferroni correction for multiple comparisons. NAV: normal aortic valve; AVSc: aortic valve sclerosis; AVSt: aortic valve stenosis. Δ: difference between values obtained at the supine position minus values at active standing. meanNN: mean value of all NN intervals (RR intervals from sinus rhythm), SDNN: standard deviation of all NN intervals; RMSSD: root mean squared of the successive differences; pNN20: percentage of successive NN intervals with differences greater than 20 ms; LF: low-frequency band spectral power; HF: high-frequency band spectral power; HFn: HF in normalized units; LFn: LF in normalized units; LF/HF: ratio between low-frequency and high frequency band indices; α_1_: short-term scaling exponent; SampEn: sample entropy.

**Table 5:** Results of the hierarchical partitioning analysis for each independent variable (indicated as HRV in the first column). The name of the independent variables and its independent contribution to the R² of the hierarchical partitioning combined final analysis are shown in columns 2 and 3. The models where there were no valve parameter function-independent variables included are indicated as dashed lines (----). The column 4 shows the pre-selected covariables included in the hierarchical partitioning analysis, as previously selected by applying multiple linear stepwise regression models for each dependent variable (see Section 2.8). The last column provides the R^2^ of the combined model assessed by the hierarchical partitioning analysis.

HRV	Valve Function Parameter (Independent Variable)		Pre-Selected Covariables	R^2^ of Combined Model
	Name	% Independent Exploratory Capacity		
ΔpNN20	PGmean	3.951	age, albumin, CRP, medication intake, ΔmeanNN	0.2890
ΔmeanNN	PGmean	2.519	LVMi, glucose, tri glycerides, ET1, age, medication intake, MBF	0.2525
ΔRMSSD	----	----	age, medication intake, ΔmeanNN	0.2966
ΔSDNN	----	----	CRP, IFN-γ, BMI, medication intake	0.2102
ΔLF	----	----	RWT, CRP, TIMP1, BMI	0.2439
ΔHF	PGmean	1.698	SBP, ΔmeanNN	0.2244
ΔLF/HF	PGmax	1.109	PGmax, RWT, SBP, medication intake, MBF, ΔmeanNN	0.1643
ΔHFn	PGmean	2.432	LVM, glucose, triglycerides, ET1, SBP, DBP, MBF, ΔmeanNN	0.3077
ΔLFn	PGmean	2.390	LVM, glucose, triglycerides, ET1, SBP, DBP, MBF, ΔmeanNN	0.3005
Δα_1_	AVA	4.591	RWT, LVEF, triglycerides, ET1, IL-4, SBP, age, medication intake, MBF, ΔmeanNN	0.2960
ΔSampEn	PGmean	0.985	MMP2/TIMP1, SBP, ΔmeanNN	0.1717

Δ: difference between values obtained at the supine position minus values at active standing; MeanNN: mean value of all NN intervals (RR intervals from sinus rhythm), SDNN: standard deviation of all NN intervals; RMSSD: root mean squared of the successive differences; pNN20: percentage of successive NN intervals with differences greater than 20 ms; LF: low-frequency band spectral power; HF: high-frequency band spectral power; HFn: HF in normalized units; LFn: LF in normalized units; LF/HF: ratio between low-frequency and high frequency band indices; α_1_: short-term scaling exponent; SampEn: sample entropy; LVEF: left ventricular ejection fraction; LVM: left ventricular mass; LVMi: left ventricular mass indexed to body surface area; RWT: relative wall thickness. Vmax: maximum aortic valve transvalvular velocity; PGmean: mean pressure gradient; PGmax: maximum pressure gradient; AVA: aortic valve area; BMI: body mass index; SBP: systolic blood pressure; DBP: diastolic blood pressure; MBF: mean breathing frequency; IL-4: interleukin 4; IFN-γ: interferon gamma; MMP2: matrix metalloproteinase 2; TIMP1: tissue inhibitors of metalloproteinases 1; ET1: endothelin 1; CRP: C-reactive protein.

## Data Availability

The data presented in this study are available on request from the corresponding author.

## Supplementary Materials

The following supporting information can be downloaded at: https://www.mdpi.com/article/10.3390/jcm11164771/s1, Table S1. Variables chosen with the multiple linear stepwise regression models analysis to be entered in the hierarchical partitioning analysis for the time domain HRV parameters. The first column shows the names of independent variables and covariables that were considered. The other columns show the variables chosen within each group of independent variables and covariables; Table S2. Variables chosen with the multiple linear stepwise regression model analysis to be entered in the hierarchical partitioning analysis for the frequency domain HRV parameters. The first column shows the names of independent variables and covariables that were considered. The other columns show the variables chosen within each group of independent variables and covariables; Table S3. Variables chosen with the multiple linear stepwise regression model analysis to be entered in the hierarchical partitioning analysis for the nonlinear HRV parameters. The first column shows the names of independent variables and covariables that were considered. The other columns show the variables chosen within each group of independent variables and covariables; Figure S1. Independent variable and covariables and their percentage of independent explanatory capacity for dependent variable ΔpNN20; Figure S2. Independent variable and covariables and their percentage of independent explanatory capacity for dependent variable ΔmeanNN; Figure S3. Independent variable and covariables and their percentage of independent explanatory capacity for dependent variable ΔRMSSD; Figure S4. Independent variable and covariables and their percentage of independent explanatory capacity for dependent variable ΔSDNN; Figure S5. Independent variable and covariables and their percentage of independent explanatory capacity for dependent variable ΔLF; Figure S6. Independent variable and covariables and their percentage of independent explanatory capacity for dependent variable ΔHF; Figure S7. Independent variable and covariables and their percentage of independent explanatory capacity for dependent variable ΔLF/H; Figure S8. Independent variable and covariables and their percentage of independent explanatory capacity for dependent variable ΔHFn; Figure S9. Independent variable and covariables and their percentage of independent explanatory capacity for dependent variable ΔLFn; Figure S10. Independent variable and covariables and their percentage of independent explanatory capacity for dependent variable Δ α_1_; Figure S11. Independent variableand covariables and their percentage of independent explanatory capacity for dependent variable ΔSampEn.
