# Supplementary material for: Association of the Heart Rate Variability Response to Active Standing with the Severity of Calcific Aortic Valve Disease: Novel Insights of a Neurocardiovascular Pathology
Source: J Clin Med. 2022 Aug 16;11(16):4771. doi: 10.3390/jcm11164771 (PMC9409634; doi:10.3390/jcm11164771)
Supplement: Supplementary file 1 [file jcm-11-04771-s001.zip › jcm-1810099-supplementary.pdf]

Supplementary Materials

Multiple linear stepwise regression models analysis

Table S1. Variables chosen with the multiple linear stepwise regression models analysis to be entered in the hierarchical partitioning analysis for the time domain HRV parameters. The first column shows the names of independent variables and covariables that were considered. The other columns show the variables chosen within each group of independent variables and covariables.

**Groups**	**ΔpNN20**	**ΔmeanNN**	**ΔRMSSD**	**ΔSDNN**
Valve function	PGmean	PGmean	- - - - - - -	- - - - - - -
Ventricular function	- - - - - - -	LVMi	- - - - - - -	- - - - - - -
Biochemical	Albumin	Glucose, Triglycerides	- - - - - - -	- - - - - - -
Inflammatory mediators	CPR	ET1	- - - - - - -	CPR, IFN-γ
Anti-inflammatory mediators	- - - - - - -	- - - - - - -	- - - - - - -	- - - - - - -
Lipidic inflammatory mediators	- - - - - - -	- - - - - - -	- - - - - - -	- - - - - - -
Lipidic resolving mediators	- - - - - - -	- - - - - - -	- - - - - - -	- - - - - - -
MMps	- - - - - - -	- - - - - - -	- - - - - - -	- - - - - - -
MMPs/TIMP1	- - - - - - -	- - - - - - -	- - - - - - -	- - - - - - -
Anthropometric and clinical -Numeric	Age	Age	Age	BMI
Anthropometric and clinical -Categorical	Medication Intake	Medication Intake	Medication Intake	Medication Intake
MBF	- - - - - - -	MBF	- - - - - - -	- - - - - - -
ΔmeanNN	ΔmeanNN	- - - - - - -	ΔmeanNN	- - - - - - -

MeanNN: mean value of all NN intervals, SDNN: standard deviation of all NN intervals; RMSSD: root mean squared of the successive differences; pNN20: percentage of successive NN intervals with differences greater than 20 ms; Δ: difference between values obtained at the supine position minus values at active standing; LVMi: left ventricular mass indexed to body surface area; RWT: relative wall thickness. PGmean: mean pressure gradient; AVA: aortic valve area; BMI: body mass index; MBF: mean breathing frequency; IFN-γ: interferon gamma; ET1: endothelin 1; CRP: C-reactive protein.

Table S2. Variables chosen with the multiple linear stepwise regression model analysis to be entered in the hierarchical partitioning analysis for the frequency domain HRV parameters. The first column shows the names of independent variables and covariables that were considered. The other columns show the variables chosen within each group of independent variables and covariables.

Groups	ΔLF	ΔHF	ΔLF/HF	ΔHFn	ΔLFn
Valve function	- - - - - -	PGmean	PGmax	PGmean	PGmean
Ventricular function	RWT	- - - - - - -	RWT	LVM	LVM
Biochemical	- - - - - -	- - - - - - -	- - - - - - -	Glucose, Triglycerides	Glucose, Triglycerides
Inflammatory mediators	CRP	- - - - - - -	- - - - - - -	ET1	ET1
Anti-inflammatory mediators	- - - - - -	- - - - - - -	- - - - - - -	- - - - - - -	- - - - - - -
Lipidic inflammatory mediators	- - - - - -	- - - - - - -	- - - - - - -	- - - - - - -	- - - - - - -
Lipidic resolving mediators	- - - - - -	- - - - - - -	- - - - - - -	- - - - - - -	- - - - - - -
MMps	TIMP1	- - - - - - -	- - - - - - -	- - - - - - -	- - - - - - -
MMPs/TIMP1	- - - - - -	- - - - - - -	- - - - - - -	- - - - - - -	- - - - - - -
Anthropometric and clinical -Numeric	BMI	SBP	SBP	SBP, DBP	SBP, DBP
Anthropometric and clinical -Categorical	- - - - - -	- - - - - - -	Medication Intake	- - - - - - -	- - - - - - -
MBF	- - - - - -	- - - - - - -	MBF	MBF	MBF
ΔmeanNN	- - - - - -	ΔmeanNN	ΔmeanNN	ΔmeanNN	ΔmeanNN

LF: low-frequency band spectral power; HF: high-frequency band spectral power; HFn: HF in normalized units; LFn: LF in normalized units; LF/HF: ratio between low-frequency and high frequency band indices; Δ: difference between values obtained at the supine position minus values at active standing; LVM: left ventricular mass; RWT: relative wall thickness. PGmean: mean pressure gradient; PGmax: maximum pressure gradient; BMI: body mass index; SBP: systolic blood pressure; DBP: diastolic blood pressure; MBF: mean breathing frequency; ET1: endothelin 1; CRP: C-reactive protein.

Table S3. Variables chosen with the multiple linear stepwise regression model analysis to be entered in the hierarchical partitioning analysis for the nonlinear HRV parameters. The first column shows the names of independent variables and covariables that were considered. The other columns show the variables chosen within each group of independent variables and covariables.

Groups	Δa1	ΔSampEn
Valve function	AVA	PGmean
Ventricular function	RWT, LVEF	- - - - - - -
Biochemical	Triglycerides	- - - - - - -
Inflammatory mediators	ET1	- - - - - - -
Anti-inflammatory mediators	IL-4	- - - - - - -
Lipidic inflammatory mediators	- - - - - - -	- - - - - - -
Lipidic resolving mediators	- - - - - - -	- - - - - - -
MMps	- - - - - - -	- - - - - - -
MMPs/TIMP1	- - - - - - -	MMP2/TIMP1
Anthropometric and clinical -Numeric	SBP, Age	SBP
Anthropometric and clinical -Categorical	Medication intake	- - - - - - -
MBF	MBF	- - - - - - -
ΔmeanNN	ΔmeanNN	ΔmeanNN

$\alpha 1$: short-term scaling index; SampEn: sample entropy; Δ: difference between values obtained at the supine position minus values at active standing; LVEF: left ventricular ejection fraction; RWT: relative wall thickness. PGmean: mean pressure gradient; AVA: aortic valve area; BMI: body mass index; SBP: systolic blood pressure; MBF: mean breathing frequency; IL-4: interleukin 4; MMP2: Matrix metalloproteinase 2; TIMP1: tissue inhibitors of metalloproteinases 1; ET1: endothelin 1.

Hierarchical Partitioning

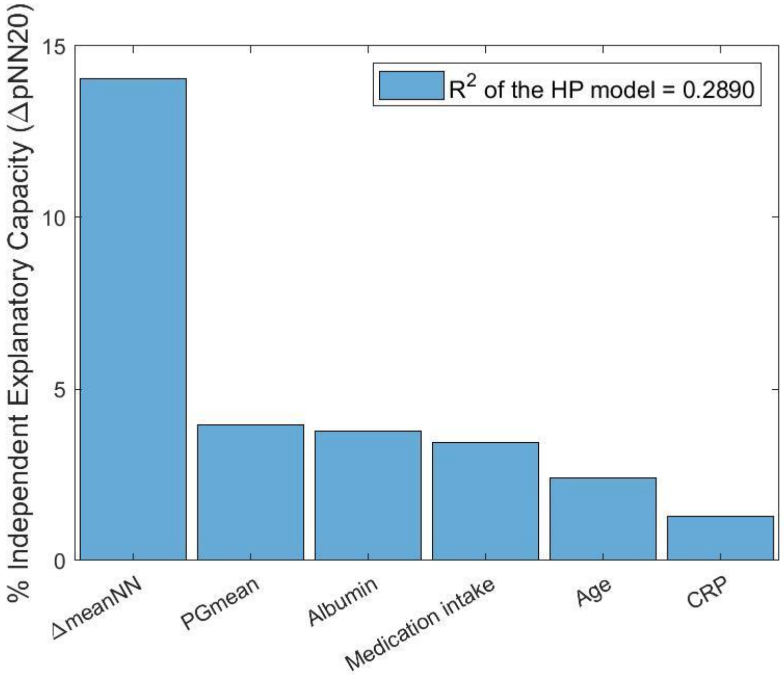

Figure S1. Independent variable and covariables and their percentage of independent explanatory capacity for dependent variable ΔpNN20.

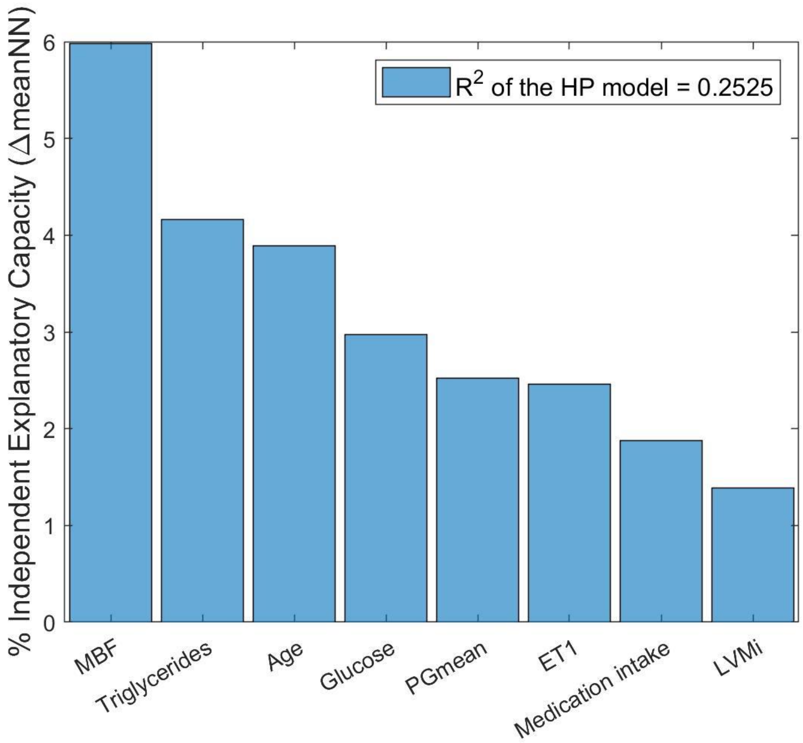

Figure S2. Independent variable and covariables and their percentage of independent explanatory capacity for dependent variable ΔmeanNN.

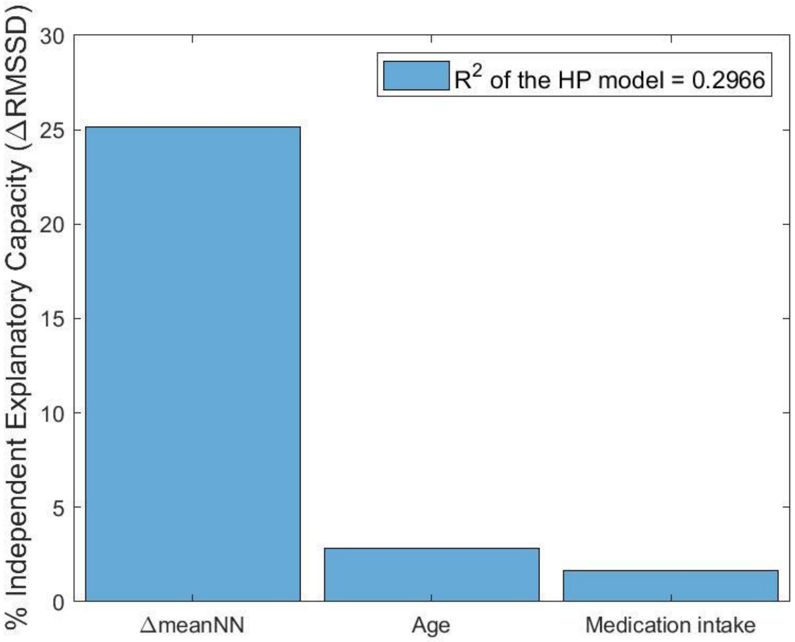

Figure S3. Independent variable and covariables and their percentage of independent explanatory capacity for dependent variable ΔRMSSD.

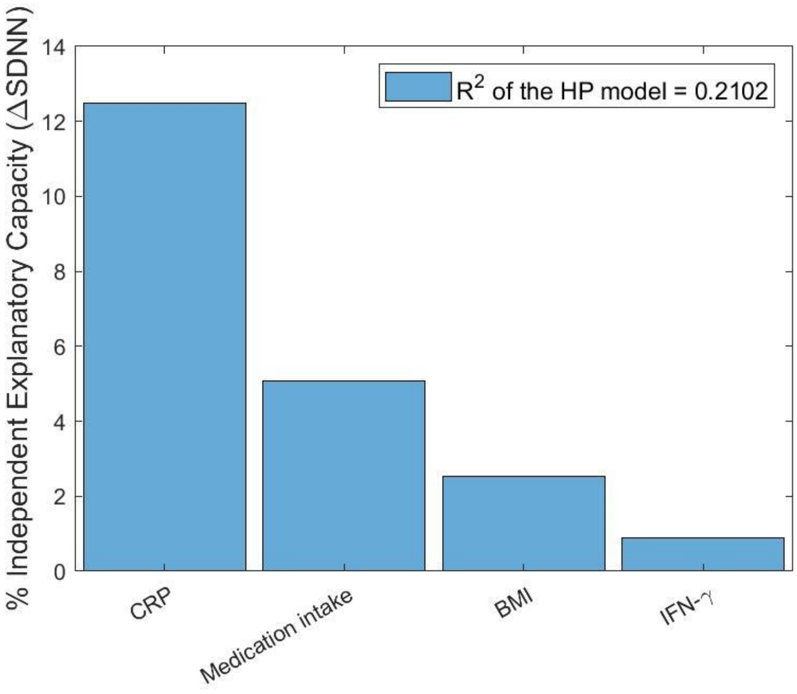

Figure S4. Independent variable and covariables and their percentage of independent explanatory capacity for dependent variable ΔSDNN.

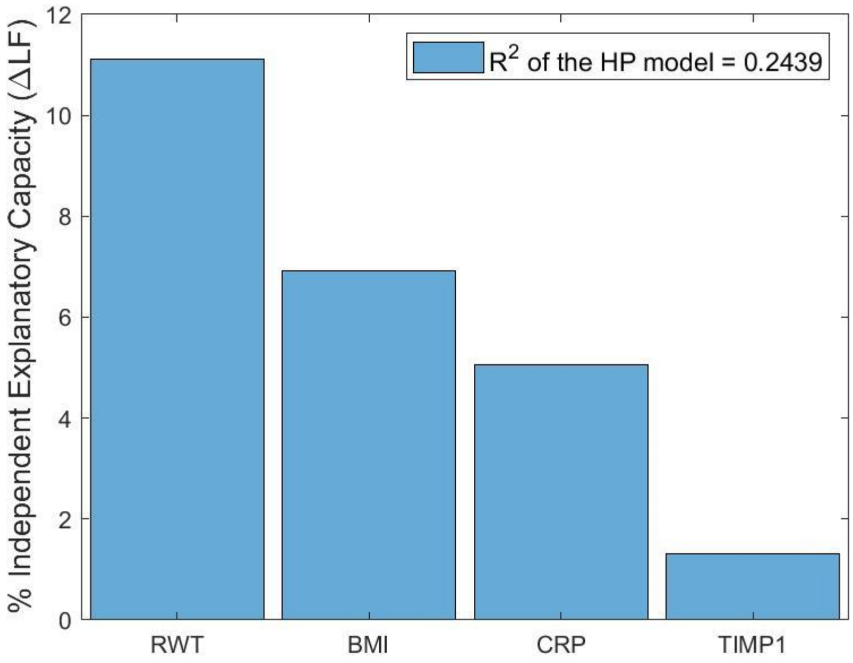

Figure S5. Independent variable and covariables and their percentage of independent explanatory capacity for dependent variable ΔLF.

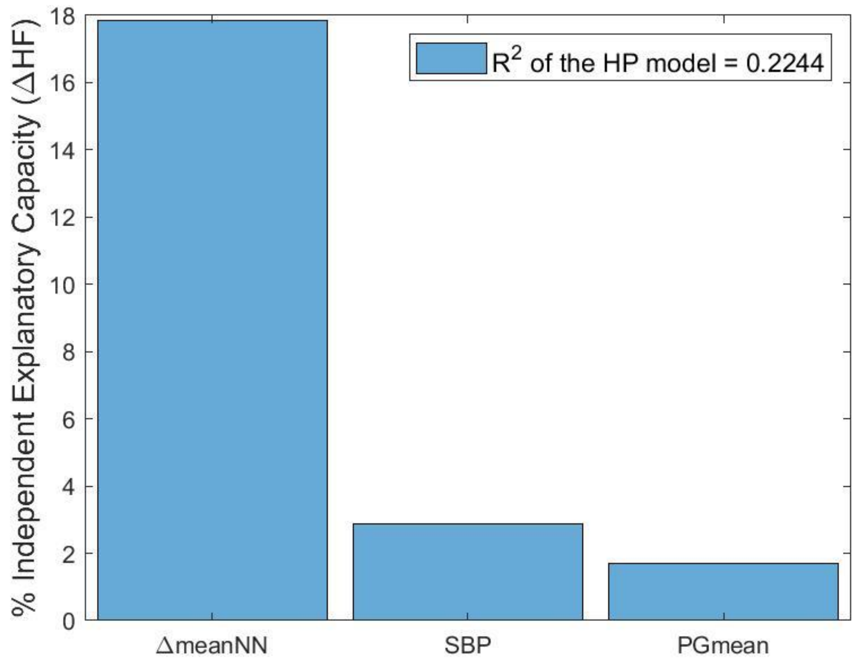

Figure S6. Independent variable and covariables and their percentage of independent explanatory capacity for dependent variable ΔHF.

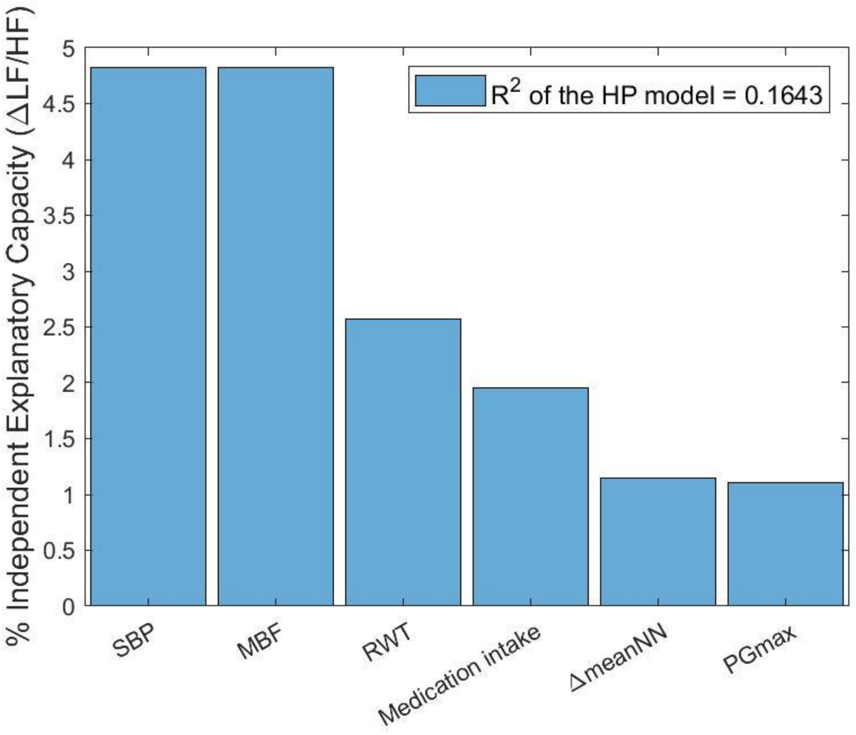

Figure S7. Independent variable and covariables and their percentage of independent explanatory capacity for dependent variable ΔLF/HF.

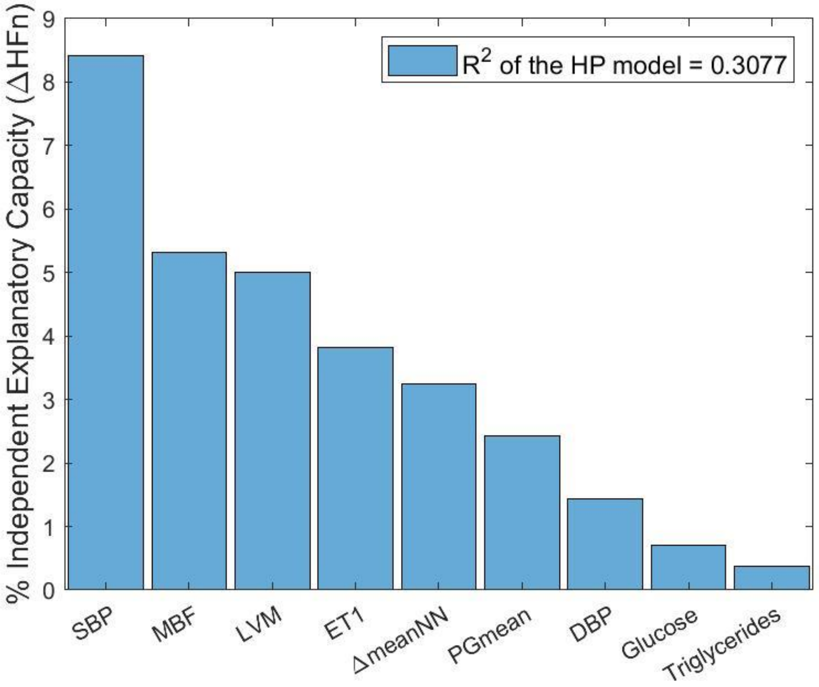

Figure S8. Independent variable and covariables and their percentage of independent explanatory capacity for dependent variable ΔHFn.

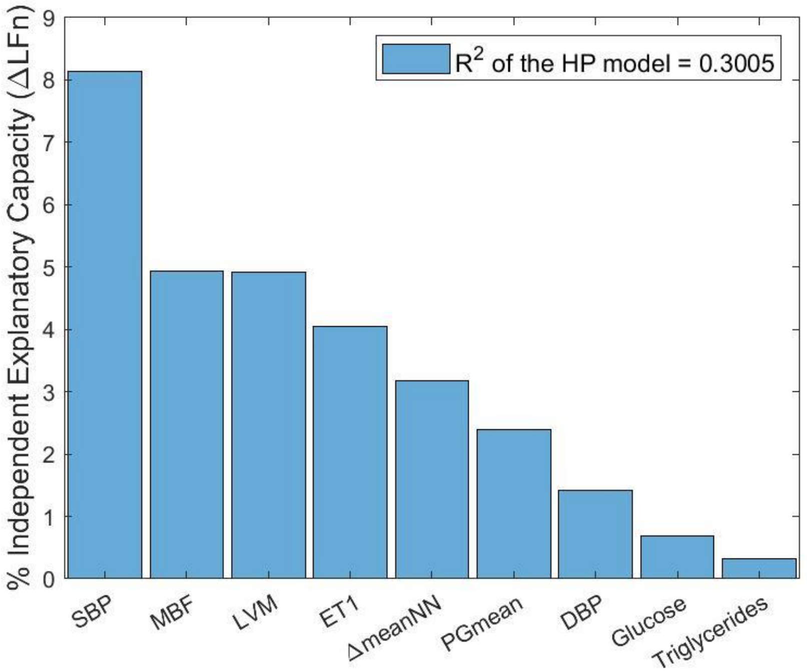

Figure S9. Independent variable and covariables and their percentage of independent explanatory capacity for dependent variable ΔLFn.

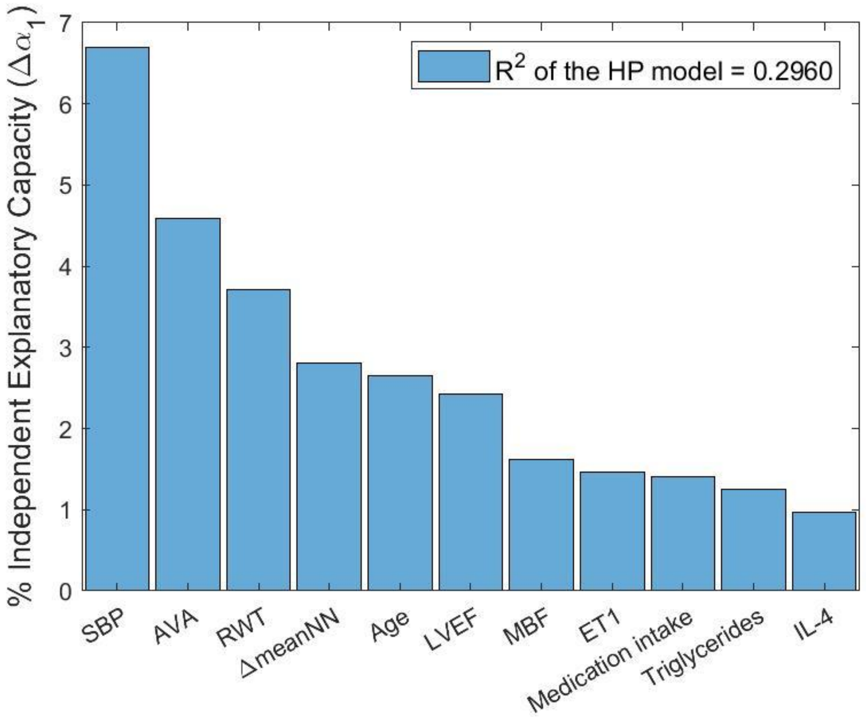

Figure S10. Independent variable and covariables and their percentage of independent explanatory capacity for dependent variable $\Delta\ \alpha_1$.

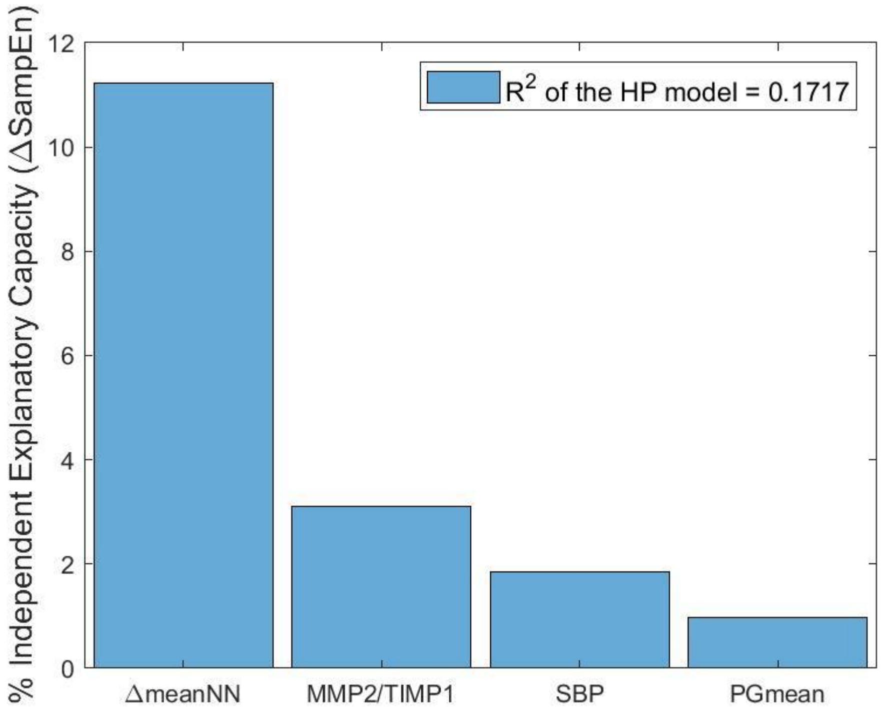

Figure S11. Independent variable and covariables and their percentage of independent explanatory capacity for dependent variable ΔSampEn.
